# Longitudinal change in the serology of antibodies to *Chlamydia trachomatis* pgp3 in children residing in a trachoma area

**DOI:** 10.1038/s41598-018-21127-0

**Published:** 2018-02-23

**Authors:** Sheila K. West, Beatriz Munoz, Hemjot Kaur, Laura Dize, Harran Mkocha, Charlotte A. Gaydos, Thomas C. Quinn

**Affiliations:** 1 Johns Hopkins School of Medicine, Ophthalmology Department, Baltimore, 21287 USA; 2 Kongwa Trachoma Project, Kongwa, Tanzania; 3 National Institute of Allergy and Infectious Diseases, Bethesda, 20814 USA

## Abstract

A serologic test for antibodies to chlamydial antigen pgp3 may be a useful tool for trachoma surveillance. However, little is known about the stability of antibody status over time, or factors associated with seroreversion/conversion. A cohort of 2,111 children ages 1–9 years in Tanzania were followed for one year in the absence of mass azithromycin. At baseline and follow-up, they were evaluated for trachoma, chlamydial infection, and antibodies to chlamydial antigen pgp3. At baseline, 31% of children were seropositive for pgp3 antibodies and 6.4% seroreverted to negative over one year. Of those seronegative, 9.8% seroconverted over the year. The seroreverters had lower baseline mean fluorescence intensity (MFI-BG) values compared to the seropositives who remained positive (Odds Ratio = 0.04 for every unit increase in log_10_MFI-BG, 95% CI = 0.02–0.09), and were more likely to live in communities with trachoma <5% (p < 0.008). While seroconversion was expected, seroreversion was unexpected. The low seroprevalence rate reported from low endemic areas may be due to seroreversion as well as lack of exposure.

## Introduction

Trachoma, a chronic conjunctivitis caused by *C*. *trachomatis*, is the leading infectious cause of blindness world-wide, although impressive strides towards elimination are being achieved^1^. The World Health Organization (WHO) has set a goal of elimination of blinding trachoma as a public health problem by the year 2020^2^. As more endemic districts within countries achieve the elimination goal of follicular trachoma (TF) prevalence <5% in children ages 1–9 years, they face the task of surveillance for potential re-emergence. Currently, the WHO recommends a surveillance survey at least two years after cessation of mass drug administration (MDA), which would consist of a prevalence survey for TF among 1–9 year olds in the district to re-confirm <5% level of TF^3^. The concern is to be certain that disease has not re-emerged.

However, surveillance surveys occur in the context of low prevalence of trachoma and there are concerns with the precision of grading TF when the disease prevalence is low. For example, the implications if the surveillance survey finds TF at 6% include the potential of having to re-start programs. Data from villages where TF is above 5% (but still low) in the absence of infection with *C*. *trachomatis* have been reported, raising concerns for over grading^4,5^. At the same time, a test of infection for *C*. *trachomatis* as the indicator for a surveillance survey is also problematic. Districts where trachoma is less than 5% for four or more years have also reported the presence of infection^6^ at the time of surveillance, suggesting that some level of infection could be tolerated and disease does not re-emerge. In other situations, notably under antibiotic pressure, communities can achieve virtually zero prevalence of infection with rates of TF above 20% and re-emergence of infection has occurred^7^. The problem with the use of TF and/or a test of infection, when assessed cross sectionally in surveys, is they provide only snapshots of the current prevalence but limited information about ongoing transmission or risk of re-emergence. For this reason, further work on additional surveillance tools that may provide more information has been recommended^3^.

The use of serology, in particular seropositivity for antibodies to chlamydial pgp3 antigen, is one potential tool. From research in ocular chlamydia conducted thus far, seropositivity appears to reflect past exposure to infection, and low or absent seropositivity seems to reflect absence of ongoing transmission^8^. Previous research in a hyper-endemic area has shown that seropositivity to Chlamydial antigen pgp3 remains high, even after MDA, with no seroreversion six months after MDA^9^. A surveillance survey in a formerly endemic district in Tanzania found TF <5%, evidence of infection at 1%, and low rates of antibody seropositivity clustered in some villages^6^. In that study, the age specific prevalence of seropositivity increased but at a very modest rate. In a surveillance survey in two districts in Nepal, TF and infection were virtually absent and the low antibody seropositivity rate, average 2%, showed no increase with age among 1–9 year olds^10^. Similar findings from cross sectional studies in villages have also been reported^4,11^. The value for using serology as a surveillance tool is the potential ability to assess cumulative exposure to transmission of ocular C. *trachomatis* over the two year period between the final prevalence survey and the surveillance survey, plus the potential for integration with tests for antigens of other neglected tropical diseases.

While the use of serologic tests for antibodies to *C*. *trachomatis* is a promising tool, further work on understanding the longevity of seropositivity and factors that affect seroconversion is needed. There have been no longitudinal studies of children in low prevalence settings to provide data on possible seroreversion as well as seroconversion, nor is it clear the number of infections required to develop seropositivity. The aims of this study are to determine the rates of seroconversion, and seroreversion (if any), in connection with trachoma and infection in a random sample of children age 1–9 years over a one year period in 50 communities in Kongwa Tanzania, where trachoma was formerly hyper-endemic.

## Methods

### Population and study cohort

Kongwa district in Tanzania was a trachoma hyperendemic area whose prevalence of trachoma decreased to <10% by 2013^12^. In April-June 2015, a random sample of 51 children ages 1–9 years in each of 52 communities that were enrolled in a clinical trial of surveillance strategies^12^ was selected for survey for that trial. The random selection of children was based on a complete census, which included age and gender, of all residents of the communities. The survey consisted of a clinical determination of trachoma, a test for infection, and a dried blood spot to test for antibodies to chlamydial antigen pgp3; these methods are described further below. We received funding to follow up that sample one year later, but two communities no longer wished to participate. In all other communities, the same children were eligible for follow up to determine the change in infection and trachoma. They were also surveyed to determine antibody status at follow up. The longitudinal cohort is defined as those who had a baseline and one year survey. During the one year prior to follow up, no mass drug administration with azithromycin was carried out.

### Survey

A trained trachoma grader, using a flashlight and 2·5 loupes, assessed each eyelid for the presence or absence of TF and trachoma inflammation-intense (TI) using the WHO simplified grading scheme^13^. For quality control purposes, a photograph, taken of the upper tarsus of the right eye of every 5th child plus all children with trachoma, ensured at least 50 photographs for purposes of monitoring any drift in trachoma grading over time. A handheld Nikon D-series camera (D-40) with a 105 mm f/2·8D AF Macro Nikkor Autofocus Lens (fully extended, in manual setting) was used. There was no evidence of drift in grading.

Following a strict protocol to avoid field contamination, a swab was taken of the left eye of every child, stored dry in a refrigerator for up to 30 days, shipped to Johns Hopkins University International Chlamydia laboratory, where it was stored at −80° ${\rm{u}}$ntil processed. In addition, a negative field control (“blue air swab”) was taken on a randomly chosen 5% sample to monitor contamination. For each negative field control, the examiner passed a sterile Dacron swab within 1 inch of the individual’s conjunctiva, and these were labeled and processed identically to true conjunctival swabs. The laboratory personnel were masked to field control and regular swabs.

Blood was taken from a single finger prick, filled up to six circular extensions which are calibrated to hold 10 µl of blood. The blood spots were dried, stored in a freezer until shipped to the International Chlamydia Laboratory at Johns Hopkins and processed for antibodies to pgp3 on Luminex 100 platform.

### Laboratory processing

The swabs were processed using a published pooling strategy^14,15^ (4–5 per pool) within 90 days of arrival, using the APTIMA Combo 2 (AC2) commercial test for *C*. *trachomatis* (Hologic Inc., San Diego CA). The pool results were recorded as positive or negative for *C*. *trachomatis*, equivocal, or invalid. For pools that yielded a negative result, all specimens in that pool were considered to be negative for *C*. *trachomatis*. For each pool that yielded a positive result, the original samples were retested in order to determine which sample (s) had a positive result. Equivocal/invalid pools were retested as well as the individual samples contained in that pool, to determine a final positive or negative result.

Details of the processing for the dried blood spots are described elsewhere^8,10^ and summarized here. Total IgG was detected using biotinylated mouse anti-human total IgG (clone H2; Southern Biotech, Birmingham, AL), IgG4 (clone HP6025; Invitrogen, South San Francisco, CA), and R-phycoerythrin-labeled streptavidin (Invitrogen, South San Francisco, CA). After washing, beads were read on a BioPlex 100 instrument (Bio-Rad, Hercules, CA) using Bio-Plex Manager 6.0 software (Bio-Rad). The level of fluorescence from each sample was reported as the median fluorescence intensity minus background intensity (MFI-BG) where background is the intensity of beads with buffer only. Antibody positivity was determined from a cut-off using ROC analyses^8^ The sensitivity and specificity of the antibody test has been reported elsewhere^8^ using sera from children who were positive for both infection and trachoma, and a US sample of children who were never exposed. The first set of beads was only sufficient to analyze the baseline data; we received additional funding for the follow up and another bead set was made for blood spots from this visit. There are two cut-offs for positivity: the cut-off value for positivity was 575 (log MFI-BG = 2.76) at baseline and 989 (log MFI-BG = 2.99) at one year.

### Data analyses

The distribution of log MFI-BG at baseline and follow up are presented, showing the cut-off values for positivity.

We divided the cohort into four groups according to their baseline and follow up antibody status: Group 1 was seropositive for pgp3 antibodies at both surveys, Group 2 was seropositive at baseline and seroreverted to negative at follow up, Group 3 was negative at baseline and seroconverted to positive at follow up, and Group 4 was seronegative at both surveys. Contingency tables were used to compare the age distribution of children in group 1 with the age distribution of children in each of the other three groups. The chi-square test was used to test for significant differences. Alternative cut-off analyses were done using two other approaches. The first was to minimize the distance between the ROC plot and the point 0,1^16^ and the second was to identify two data clusters corresponding to negative and positive samples using the K-means clustering algorithm with k = 2^16^. Data were re-analyzed using these approaches for changes in antibody status. Multi-variate logistic regression models were used to examine the contribution of baseline and follow-up MFI-BG levels to rates of seroconversion and seroreversion while adjusting for age. A t-test for two independent samples was used to examine the association between baseline prevalence of trachoma and rates of seroreversion and seroconversion.

All procedures conformed to guidelines and regulations published and approved by the Johns Hopkins Institutional Review Board and the Tanzania Institute for Medical Research. In accordance with tenets set forth in the Declaration of Helsinki written, informed consent was obtained from the guardians of each child in the research project, and assent obtained for children over age 7 years.

### Data availability

The datasets generated during and/or analyzed during the current study are available from the corresponding author on reasonable request.

## Results

A total of 2536 children were surveyed at baseline, and 83% (2111) were followed up one year later. There was no difference by age, gender, infection status at baseline, trachoma status at baseline, or antibody positivity at baseline for those who were not followed up and those in the follow up survey (Table 1). At baseline, the TF prevalence in all children in the baseline survey was 5.2% and the prevalence of infection was 4.9%. None of the air swab controls were positive, supporting an absence of contamination in the field and in the laboratory.

**Table 1:** Baseline characteristics of children age 1–9 years who were followed at one year compared to those not followed at one year.

Baseline characteristic	Longitudinal cohort n = 2111	Lost to follow up at one year n = 425	p- value
Age in years (Mean (SD))	4.42 (2.63)	4.29 (2.72)	0.38
Female (n (%))	1070 (50.7)	210 (49.5)	0.66
Follicular Trachoma (TF) (n (%))	109 (5.2)	23 (5.4)	0.83
Infection (n (%))	97 (4.6)	28 (6.6)	0.08
Antibody positive (n (%))	657 (31.1)	143 (33.7)	0.31

**Figure 1:**
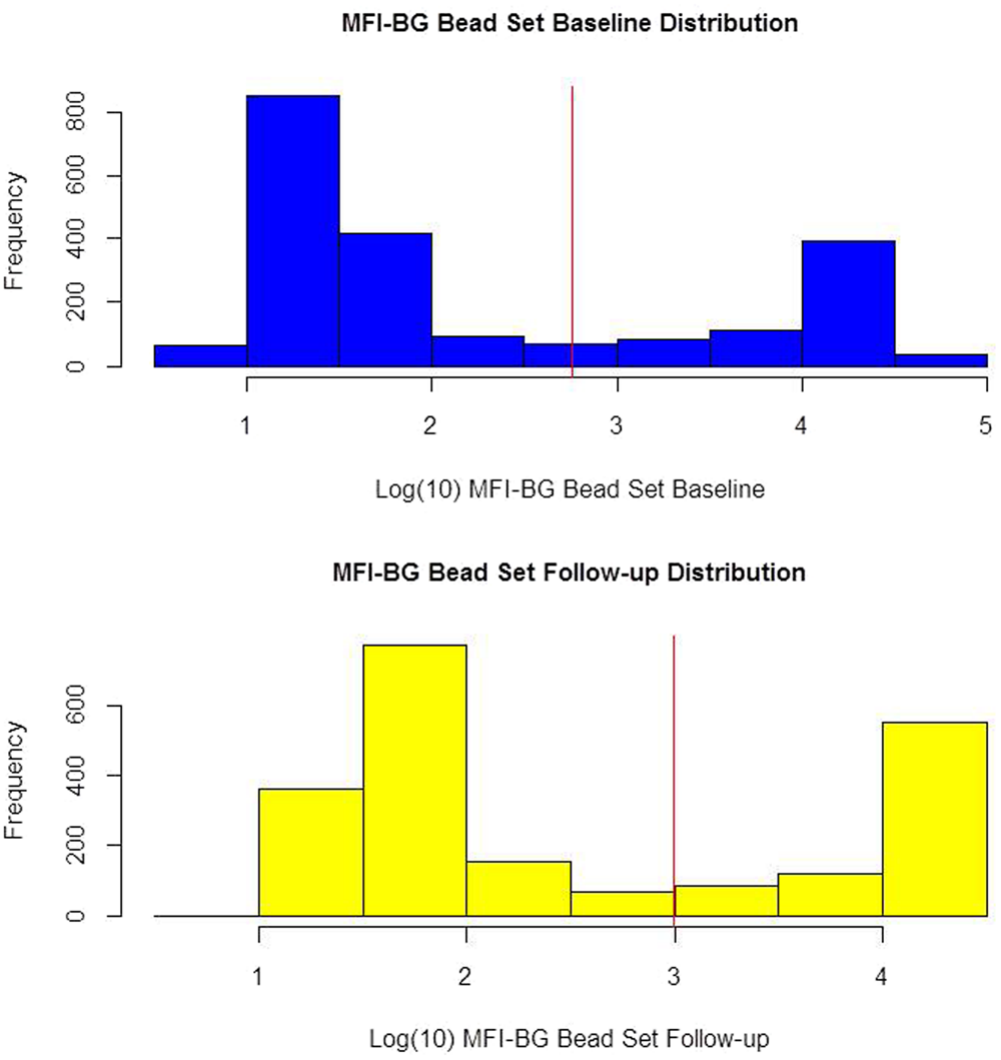
Log_10_ of MFI-BG at baseline and at follow up for the longitudinal cohort, with the red line showing the cut-off for positivity of each distribution.

Figure 1 shows the distribution of the log_10_ MFI-BG at baseline and follow up, suggesting two distributions for each time point.

For the 2111 children in the longitudinal cohort, 31.1% were seropositive for antibodies at baseline. Of those who were seropositive at baseline, 42 (6.4%) seroreverted to negative after one year. Of those who were negative at baseline, 143 (9.8%) seroconverted to positive at one year (Table 2).

**Table 2:** Change in antibody status over time in longitudinal cohort of children ages 1–9 years.

Antibody Status at baseline	Antibody status at one year follow up		Total	
	Negative	Positive		
Negative	1311	143	1465	Seroconversion rate = 9.8%
Positive	42	615	656	Seroreversion rate = 6.4%

**Figure 2:**
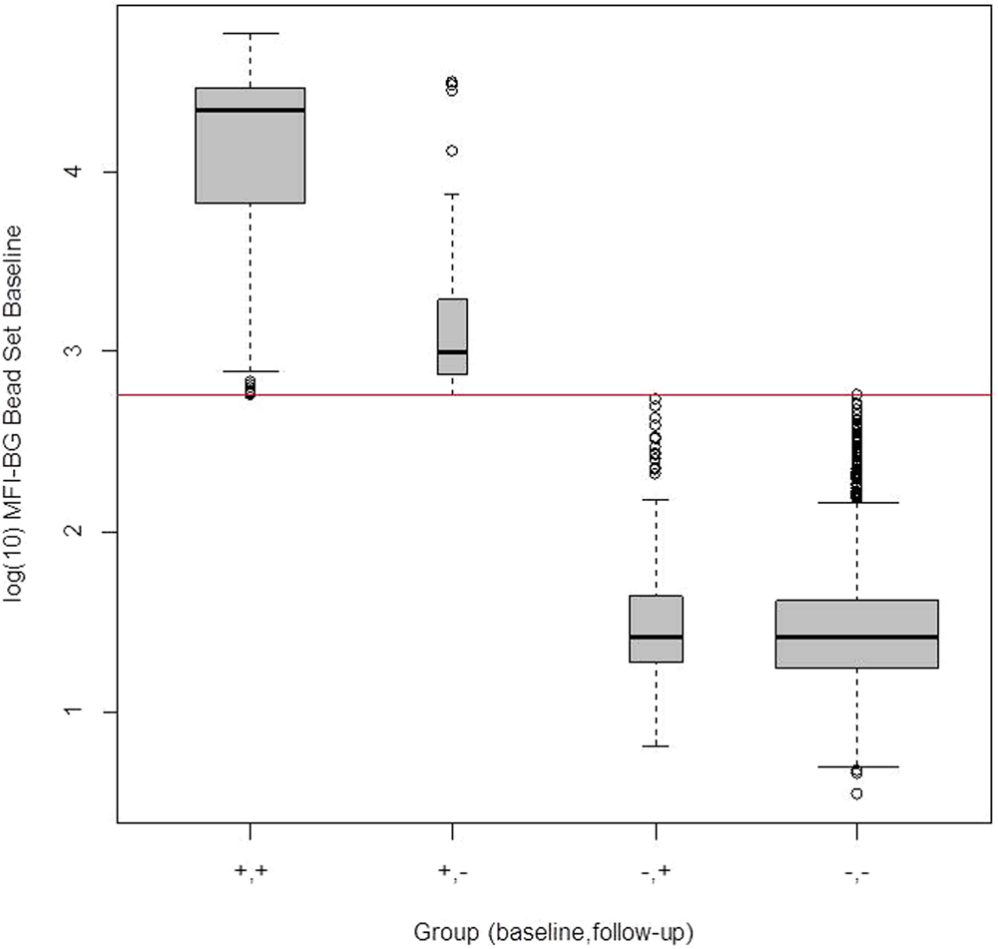
Log_10_MFI-BG at baseline for the longitudinal cohort within the four groups according to antibody status at baseline and follow up.

**Figure 3:**
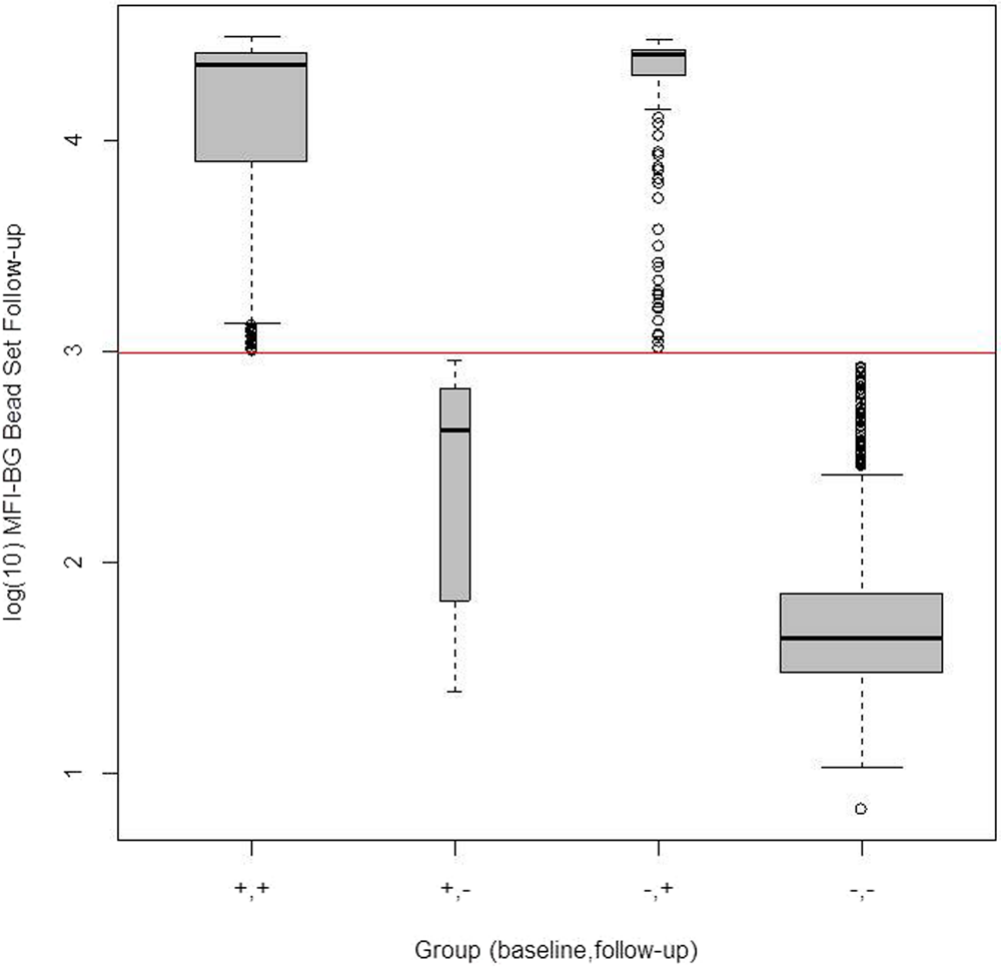
Log_10_MFI-BG at one year follow up for the longitudinal cohort within the four groups according to antibody status at baseline and follow up.

Among those who were seropositive at baseline, the group who seroreverted had a significantly lower baseline average MFI-BG than those who stayed positive at one year (p < 0.0001) (Fig. 2); the odds ratio from a multi-variate model (controlling for age) predicting seroreversion was 0.04 for every unit increase in baseline log_10_ MFI-BG (95% confidence interval = 0.02 to 0.09). Among those who were seronegative at baseline, the group who seroconverted had slightly higher average log_10_MFI-BG compared to those who stayed negative at one year, but the difference was not statistically significant. (p = 0.08). Figure 3 shows the log_10_MFI-BG at follow up for each of the 4 groups. The groups who were seropositive at follow up (which include the baseline seronegative converters) have similar average MFI-BG values. However, among the seronegative group at follow up, the seroreverters had significantly higher average log_10_MFI-BG value compared to those who were seronegative at baseline (p < 0.0001); the odds ratio for seroreversion was 30.8 for every unit increase in follow up log_10_MFI-BG (95% CI = 14.7 to 64.5).

These results suggest that some of the seroreversion may be due to the selection of cut-off values for antibody positivity, which were based on the protocol of Goodhew *et al*.^8^ using external standards. We also evaluated the cohort using alternative cut-off approaches, which resulted in minor changes to the cut-off values (Table 3) However, the results did not change.

**Table 3:** Rates of Seroreversion and Seroconversion when the cut-off values for antibody positivity are selected using different methods.

	Group 1: + /+	Group 2: + /−	Group 3: −/+	Group 4: −/−
Minimum Distance between Sensitivity and specificity, using result values	615	49	141	1306
Using K-means (k = 2) to identify the two clusters of negative and positive samples	615	42	143	1311
Original (Take average of results points closest to shoulder of ROC curve)	615	42	143	1311

The children who remained seronegative were more likely to be the youngest age group, ages 1–3 years, compared to those who were seropositive at both time points(p < 0.0001); the latter were more likely to be ages 7–9 (Table 4). Seroreversion was not different by age group.

**Table 4:** Percentage within each age group who seroconverted, seroreverted or remained the same at one year.

Antibody groups	Group 1: +/+	Group 2: +/−	Group 3: −/+	Group 4: −/−	Total
Ages					
1–3	13.5% (113)	2.0% (17)	8.4% (70)	76.1% (636)	100% (836)
4–6	31.5% (228)	2.1% (15)	7.5% (54)	58.9% (426)	100% (723)
7–9	49.6% (274)	1.8% (10)	3.4% (19)	45.1% (249)	100% (554)

Group 1 vs group 2: p = 0.001. Group 1 vs group 3: p < 0.0001.

Group 1 vs group 4: p < 0.0001.

We examined the relationship between infection at baseline and one year, and serology (Table 5). At baseline, there were 9 infections in 1453 seronegative children; all had seroconverted at one year. In the 657 seropositive children at baseline, there were 88 infections. All but two children remained seropositive. These two cases had MFI-BG values at baseline of 31,520 and 30,528 respectively, which were very positive; at follow up, the MFI-BG values were 25 and 68, respectively, well below the cut-off of 998 MFI-BG. At one year, there were 159 infections, all but 7 occurred in those children who were seropositive at one year. Those 7 children were seronegative for antibody to pgp3 at both time points.

**Table 5:** Serologic status and infection status at baseline, and one year serologic status.

Baseline serologic status	Seronegative				Seropositive			
n	1454				657			
Baseline infection status	Infection positive		Infection negative		Infection Positive		Infection Negative	
n	9		1445		88		569	
One year serologic status	Sero Positive	Sero negative	Sero positive	Sero negative	Sero positive	Sero negative	Sero positive	Sero negative
n	9	0	134	1311	86	2	529	40

Table 6 shows the seroconversion and reversion rates in communities according to their baseline trachoma status. Almost all the seroreversion occurs in communities where the TF rate is <5% (p = 0.008), where most of the seroconversion occurred where TF >5%.

**Table 6:** Community level prevalence of follicular trachoma in 1–9 year olds, and average rates of seroconversion and seroreversion.

Prevalence of TF in 1–9 year olds at baseline (community level)	Number of communities	Average yearly rate of Seroconversion	Average yearly rate of Seroreversion
0%	2	1.5	18.8
1–4%	26	10.3	12.2
5–9%	16	11.2	3.7
10%+	6	6.6	1.5
<5%	28	9.7*	12.6**
≥5%	22	10.0*	3.1**

*t-test p = 0.92, **t-test p = 0.008.

## Discussion

This large cohort study of children in a low trachoma endemic district were followed for rates of seroconversion or seroreversion over a one year period. There was good evidence for seroconversion, estimated at about 10% of those who were seronegative at baseline. All but two cases who had infection at baseline either seroconverted or maintained seropositive status. Of note, no further MDA was provided in the interim year so the seroconversion rate reflects what would be expected in these communities over a year in the absence of MDA. These data suggest that transmission was still ongoing in these communities, even though at baseline the overall rates of disease and infection were close to 5%, with seroconversion more likely in the communities still above 5%. Seroconversion was greatest in the youngest age children, reflecting the greater at-risk pool for acquiring infection(s) in these communities. The number of infections needed to produce seroconversion cannot be ascertained from our data, as we did not sample frequently enough in the seroconverters over the year. All of the seronegative children with infection at baseline did seroconvert. We also observed 134 seroconverters in those with no infection at baseline, who likely obtained infection(s) after the baseline survey, over the course of the year before we sampled them again. Of note, however, are the two cases of infection at baseline who were already seropositive, and who clearly seroreverted by the end of the year. These cases suggest that a known infection in some children already seropositive was insufficient to maintain positivity over the course of a year.

This is the first report of seroreversion in trachoma, where seropositivity to chlamydial antigen pgp3 became negative after one year in an endemic population. A previous longitudinal study over a 6 month period following MDA in a trachoma hyperendemic community found no evidence of loss of seropositivity^9^. However, the MFI-BG levels following MDA in that study were overall lower than the levels pre-MDA. Our seroreversion rate was 6% of the children who were seropositive at baseline, in the absence of any MDA. Ideally, we would have followed the children from baseline to one year using the same bead set to analyze antibody levels so that individual changes over time could be observed. This was not possible due to funding issues. We observed that the seroreversion group as a whole had MFI-BG values close to the cut off at baseline, and seronegative values again close to the cut off at follow up, and we were concerned that some of the seroreversion could be due to the method chosen for selection of cut-off values. We tried two other methods for selecting the cut off value, but the inferences were the same. We did not use the approach that fixes the specificity at 90%, as that made no sense in the context of our data; at 90% specificity, we had virtually 100% sensitivity, but which was also obtainable with higher values of specificity. Other alternatives to selecting a cut-off point use the distribution of data itself^17^, rather than external standards, and we showed that the results are identical to our original results. However, we do not feel this is reasonable where antibody prevalence may be very high or low because it has the potential to artificially create “negative” or “positive” serology in a hunt for two distributions. While we cannot exclude the possibility that some of the seroreversion could be due to selection of cut off values, regression to the mean, and noise, there is also evidence that seroreversion was a true phenomenon in this setting, as shown by the extreme MFI-BG values in two seroreversion cases with infection at baseline, and their very low MFI-BG values at one year.

Maternal antibodies to Chlamydial antigen pgp3 would be expected in newborns born to mothers with genital chlamydia, as the test for antibodies does not distinguish between the ocular and urogenital serovars. However, maternal antibodies wane over a period of 6 to 12 months^18,19^ so decay of maternal antibodies might affect the seroreversion rate in only those age up to 1 year in this study. Of the 836 children ages 1–3 years, only 3 seroreverters were in the youngest age, suggesting only 0.3% of the 2% seroreversion in the age group 1–3 years was possibly due to waning of maternal antibodies.

Researchers who have worked with an ELISA and other tests for genital strains of chlamydia reported in a cross sectional survey a loss of seropositivity to pgp3 antigen in women with time since last documented infection^20^. While 92% of women were positive for pgp3 antigens within 6 months of a diagnosed genital infection using the ELISA assay, the proportion dropped to 71% between 6–12 months and to 64% within 1–4 years. The study was cross sectional so it is not possible to know the baseline rates at time of infection nor seroreversion rate in individuals over these periods of time. However, a drop of almost 20% in the prevalence from the group within 6 months of infection to the group between 6 and 12 months is substantial (assuming the women in the group at 6–12 months had a seropositivity status of 92% within 6 months of their infection). The decline is considerably greater than the seroreversion rate of 6% we observed over one year in children in trachoma communities. However, this difference is compatible with our finding in children who resided in villages with no trachoma, where the seroreversion rate was 19%, suggesting that lack of exposure to transmission of ocular chlamydia in these communities results in appreciable seroreversion.

Within our cohort, 91% maintained their baseline antibody status at one year, with most of the change in antibody status due to seroconversion. This suggests some stability of antibody status at least over one year in low trachoma endemic communities but where transmission may still be present. The sample was also characterized by an age prevalence curve of 15.6% seropositivity at baseline among the 1–3 year olds, 33.6% among the 4–6 year olds, and 51.3% among the 7–9 year olds, suggesting not only is this sample still experiencing transmission but had past high rates of infection. This increase in seropositivity by age was not as sharp as observed in hyperendemic areas, where virtually everyone over age 6 years was seropositive^9^. It is also in contrast to the collapse of the age specific increase in seroprevalence which has been one of the features of testing in formerly endemic districts where TF has been eliminated^6,10,11^. While some of the lack of age specific increase in seropositivity has been attributed to the lack of transmission of infection in these areas, it is possible that some of this is due to seroreversion in communities where there is low or absent trachoma. Our data suggest that a single documented infection may not be enough to maintain seropositivity.

There are limitations to the study. With only two time points in which data were collected on infection and disease, we do not have sufficient information to determine the number of infections to cause seroconversion. Data from research using an ELISA test in women with urogenital infections for antibodies to pgp3 suggested that one infection was sufficient, although underreporting of infections with genital chlamydia is a serious issue for attempting to ascertain the true number of infections^20^. In addition, the ELISA test, while the most sensitive of the tests for antibody in that study, was still only 59% sensitive compared against known infection, suggesting either an insensitive test or that in some cases, more than one infection may be needed to cause seroconversion or maintain seropositivity over time^21^. While in our study all the cases of infection at baseline who were seronegative did convert by year one, we do not know at what time in the course of the year seroconversion (or seroreversion) occurred and if more than one infection was involved. With future surveys, we will be able to determine the longevity of the antibodies detected in the seroconverters. There is the possibility of misclassification of the two children who had high MFI_BG levels at baseline co-incident with infection and sero-reverted. Although we do not have conjunctival images to be certain they were the same children, we have study procedures to minimize possible misclassification. The invitations to the survey go directly to the household and the mother brings the invitation and the requested child. We are able to check the name, age, and gender of the child directly before the exam, which should avoid the wrong child presenting. We had no other child in the families of these children who went from negative to positive at follow up, which would suggest a mix up within the family. Another limitation is the use of two different bead arrays, one at baseline and another at one year. This prohibited us from following the MFI-BG levels of antibodies in the same children over time to detect individual levels of change. Nevertheless, if trachoma control programs institute the use of seroprevalence for surveillance, they will be using cut-off analyses to determine the seroprevalence of antibodies in populations and not following specific individuals over time. Finally, our use of the multiplex bead array to detect antibodies from an ocular infection may have resulted in some false negatives, as it only detects IgG in serum, not all the antibody isotypes, many of which are more prominent but not long lasting in tears; thus, at follow up where we observed infections in those who were still seronegative may be due to absence of serum IgG to pgp3 at the time of data collection. We are aware of the impact of cut-off selection in determining the incidence of seroconversion and seroreversion, although the separation of the distributions was marked and the result of alternative selection algorithms did not affect our results.

In summary, while seroconversion to antibody positive, estimated at 9.8%/year, is to be expected in this district where trachoma is not yet eliminated, seroreversion over just one year was unexpected. While the rate is small, a fraction of the lack of age specific increase in prevalence of antibodies seen in low endemic areas may be due to seroreversion rather than lack of exposure. For example, the data from Nepal showed a lack of age increase in seropositivity^9^, but our data suggest that some of the lack of positivity in older age groups may have resulted from loss of antibody, not that antibody positivity never existed in that age group. Further follow up in longitudinal samples is warranted to determine the impact of MDA and the characteristics of those who lose seropositivity status over time.
